# Homeobox A11 hypermethylation indicates unfavorable prognosis in breast cancer

**DOI:** 10.18632/oncotarget.14216

**Published:** 2016-12-25

**Authors:** Bingshu Xia, Ming Shan, Ji Wang, Zhenbin Zhong, Jingshu Geng, Xiaohui He, Tung Vu, Dekai Zhang, Da Pang

**Affiliations:** ^1^ Department of Breast Surgery, Harbin Medical University Cancer Hospital, Harbin, 150081, China; ^2^ Department of Pathology, Harbin Medical University Cancer Hospital, Harbin, 150081, China; ^3^ Department of Medical Records, Harbin Medical University Cancer Hospital, Harbin, 150081, China; ^4^ Center for Infectious and Inflammatory Diseases, Institute of Biosciences and Technology, Texas A&M University Health Science Center, Houston, Texas, 77030, USA; ^5^ Heilongjiang Academy of Medical Sciences, Heilongjiang Province, Harbin, 150086, China

**Keywords:** breast cancer, DNA methylation, HOX genes, biomarker, prognosis

## Abstract

Homeobox A11 (HOXA11) is one of the hypermethylated genes in breast cancer and its function in breast tumorigenesis remains elusive. In this study, we analyzed the methylation status of HOXA11 in 264 paired breast cancer and normal tissue as well as in matched serum samples by MethyLight assay. Further, the function of HOXA11 in breast tumorigenesis was analyzed by cell proliferation and migration assays. We found that HOXA11 was hypermethylated in cancer tissues (45.08%), especially in invasive ductal carcinomas (P<0.001), patients with a family history of cancer (P=0.033), cases with metastatic lymph nodes (P=0.004) and P53 positive group (P=0.017). Kaplan-Meier survival analysis and Cox regression analysis revealed that HOXA11 hypermethylation is an independent predictor of poor outcomes. The over expression of HOXA11 suppressed cell growth in MDA-MB-231, MCF7, SKBR3 and BT474 cells. In conclusion, the hypermethylation of HOXA11 is an independent prognostic biomarker in breast cancer. Additionally, HOXA11 can be a potential tumor suppressor.

## INTRODUCTION

Aberrant methylation of normally unmethylated CpG islands in promoter region has been associated with transcriptional inactivation of tumor suppressor genes which frequently undergo LOH in human cancers [1]. Attenuation of tumor suppressor genes by promoter methylation is implicated in tumorigenicity. These findings encourage us to identify potential tumor suppressor genes by screening methylation status of LOH loci including chromosome 7 [2]. One of the top ranked genes is HOXA11.

HOX proteins function as transcriptional factors through homeodomain, a highly conserved DNA binding domain, and are deregulated in cancers [3] including breast cancer [4, 5]. HOXA11, as a member of HOX proteins, locates in the cluster with other contiguous HOXA genes along the short arm of chromosome 7 [6] (Supplementary Figure 1). Recently, hypermethylation of HOXA11 is uncovered in ovarian cancer [7], lung cancer [8], gastric cancer [9] and breast cancer [10, 11]. Despite the methylation patterns in cancer, the clinical significance of HOXA11 methylation and its function in breast cancer remains elusive.

In this study, we used MethyLight assay to evaluate the methylation level of HOXA11 promoter region in paired normal and cancer tissues as well as in matched serum samples, then determined whether methylation status is associated with clinicopathological features or disease prognosis. We additionally analyzed the correlation between HOXA11 methylation and its function in cell proliferation and migration.

## RESULTS

### Methylation profile of HOXA11 in breast cancer

**Figure 1:**
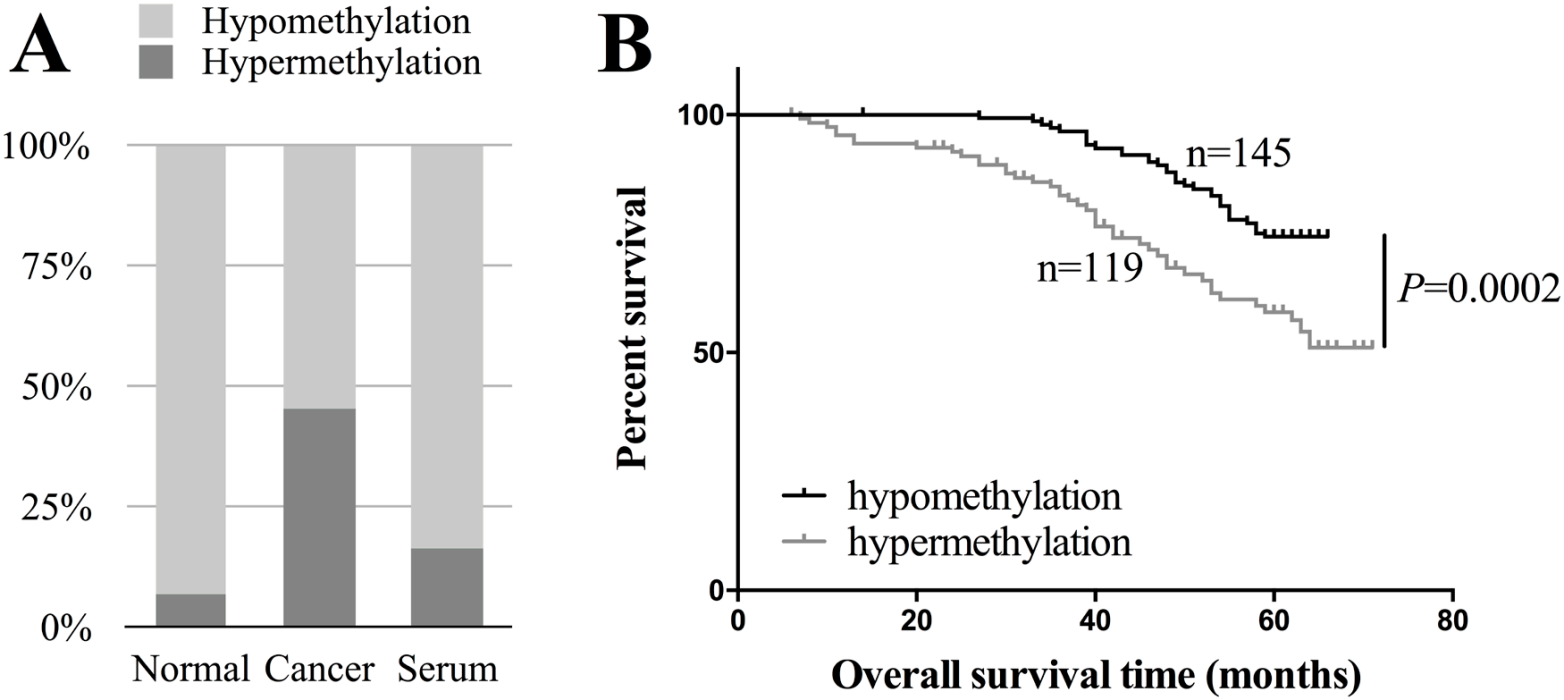
Identification of HOXA11 as a hypermethylated gene in breast cancer **A**. The methylation rates in breast cancer tissues, paired normal tissues and matched serum samples were 45.08%, 6.82% and 16.36%, respectively. **B**. Kaplan-Meier curves show that patients with hypermethylated HOXA11 have a significant worse prognosis than with hypomethylation of HOXA11 (P=0.0002, log-rank test). The cut-off value is 1.5 in MethyLight assay.

264 paired cancer and normal tissues collected after potential curative resections of breast cancers were examined by MethyLight assay using HOXA11 promoter region specific primers and probe (Supplementary Figure 1). Hypermethylation of HOXA11 represented 119 out of 264 (45.08%) primary breast tumors surveyed, while only 6.82% of normal tissues were defined as HOXA11 hypermethylation. The methylation rate (16.36%) of HOXA11 in matched serum samples was much lower than in tumors (Figure 1A).

### HOXA11 hypermethylation is a prognostic biomarker in breast cancer

We wondered whether the methylation level of HOXA11 promoter has any correlation with clinicopathological characteristics (Table 1). When comparing HOXA11 methylation level to clinical features, we noticed a significant higher incidence of hypermethylation in invasive ductal carcinomas (IDCs), patients with a family history of cancer, cases with metastatic lymph nodes and P53 positive group than their counterparts. There was no statistically significant correlation between HOXA11 methylation and other clinical pathological factors, including age, tumor size, stage, histological grade, ER status, PR status, HER2 status, Ki67 status and molecular subtypes.

**Table 1:** The correlation between HOXA11 promoter methylation and clinicopathological parameters

Characteristics		hypomethylation	hypermethylation	*P* value
Age	<50 y	89 (53.0%)	79 (47.0%)	0.399
	≥50 y	56 (58.3%)	40 (41.7%)	
Family history	no	134 (57.3%)	100 (42.7%)	0.033
	yes	11 (36.7%)	19 (63.3%)	
Pathology	DCIS	25 (96.2%)	1 (3.8%)	<0.001
	IDC	120 (50.4%)	118 (49.6%)	
Tumour size	≤2 cm	66 (52.0%)	61 (48.0%)	0.354
	>2 cm	79 (57.7%)	58 (22.0%)	
LNM	negative	72 (65.5%)	38 (34.5%)	0.004
	positive	63 (43.8%)	81 (56.3%)	
TNM Stage	0, I	38 (50.0%)	38 (50.0%)	0.306
	II, III	107 (56.9%)	81 (43.1%)	
Histological Grade	I, II	106 (55.2%)	86 (44.8%)	0.89
	III	39 (54.2%)	33 (45.8%)	
ER status	negative	57 (61.3%)	36 (38.7%)	0.126
	positive	88 (51.5%)	83 (48.5%)	
PR status	negative	62 (57.4%)	46 (42.6%)	0.502
	positive	83 (53.2%)	73 (46.8%)	
HER2 status	negative	88 (53.3%)	77 (46.7%)	0.504
	positive	57 (57.6%)	42 (42.4%)	
Ki67 status	negative	95 (57.2%)	71 (42.8%)	0.329
	positive	50 (51.0%)	48 (49.0%)	
P53 status	negative	113 (59.5%)	77 (40.5%)	0.017
	positive	32 (43.2%)	42 (56.8%)	
Molecular Subtypes	Luminal A	76 (53.1%)	67 (46.9%)	0.799
	Luminal B	25 (53.2%)	22 (46.8%)	
	HER2	27 (61.4%)	17 (38.6%)	
	TNBC	17 (56.7%)	13 (43.3%)	

Abbreviations: DCIS = ductal carcinoma *in situ*; ER = estrogen receptor; IDC = invasive ductal carcinoma; LNM = lymph node metastasis; PR = progesterone receptor; TNBC = triple negative breast cancer; TNM = tumor node metastasis.

Next, we investigated the relationship between overall survival and HOXA11 methylation. Kaplan-Meier survival analysis revealed that HOXA11 hypermethylation is a significant predictor of subsequently death (P=0.0002, Figure 1B). Both univariate (Supplementary Table 1) and multivariate (Table 2) analyses were applied to evaluate the effects of HOXA11 methylation and clinicopathological features on prognosis. In Cox proportional hazards model, HOXA11 methylation (P<0.001), tumor size (P=0.005) and histological grade (P=0.002) were significant independent predictors of poorer clinical outcome. Therefore, HOXA11 methylation is an independent prognostic factor in breast cancer.

**Table 2:** Prognostic factors in the Cox proportional hazards model

Variables	Multivariate	
	P value	95% CI
Age (years)	0.724	0.571-1.475
Tumor size (cm)	0.005	0.282-0.794
LNM	0.104	0.332-1.109
TNM Stage	0.238	0.430-1.233
Histological grade	0.002	1.626-9.154
HER2 status	0.912	0.625-1.693
HOXA11 methylation	<0.001	0.213-0.543

Abbreviations: CI = confidence interval; ER = oestrogen receptor; LNM = lymph node metastasis; PR = progesterone receptor; TNM = tumour node metastasis.

### Demethylation restored HOXA11 mRNA expression in breast cancer cell lines

**Figure 2:**
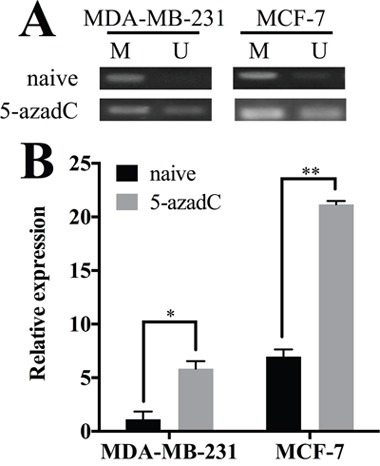
Hypermethylation of HOXA11 controls its expression in breast cancer **A**. The methylation levels of HOXA11 promoter region in MDA-MB-231 and MCF-7 cells with or without 5-azadC treatment. The methylated control was genomic DNA methylated by M.SssI and the unmethylated control was peripheral lymphocyte DNA. **B**. 5-azadC treatment restored HOXA11 expression in MDA-MB-231 (P=0.0215) and MCF-7 cells (P=0.0013). Black columns represent the expression level in cells without treatment. Gray columns represent the expression level in cells with 5-azadC treatment. Values are means ± SD, and the mean values obtained with naive MDA-MB-231 cells were set to 1. SD, standard deviation.

We employed methylation specific PCR (MSP) to determine the methylation status of HOXA11 in breast cancer cell lines. Two sets of primers targeting the CpG island near the transcription start site in the promoter region of HOXA11 [12] were employed to evaluate the methylation status in naive MCF-7 and MDA-MB-231 cells (Figure 2A). The promoter region was completely methylated in MDA-MB-231 cells and partially methylated in MCF-7 cells. Later, MCF-7 and MDA-MB-231 cells were treated with 5-aza-2’-deoxycytidine, a pharmaceutical demethylation reagent (Figure 2A). The demethylation restored the expression of HOXA11 in MDA-MB-231 (P=0.0215) and MCF-7 cells (P=0.0013) (Figure 2B). These results indicated that HOXA11 methylation correlated inversely with its expression in MDA-MB-231 and MCF-7 cells.

### High expression of HOXA11 inhibits cell proliferation

**Figure 3:**
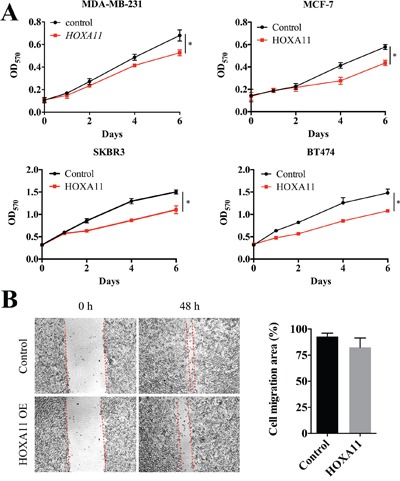
HOXA11 suppressed cell proliferation and migration in MDA-MB-231 cells **A**. The over expression of HOXA11 inhibits cell proliferation in MDA-MB-231 (P=0.0322), MCF7 (P=0.0493), SKBR3 (P=0.0409) and BT474 (P=0.0320) cells. **B**. Wound healing assay shows that the over expression of HOXA11 inhibits migration in MDA-MB-231 cells. The cell migration area was measured by ImageJ (P=0.1341).

To investigate the function of HOXA11 in carcinogenesis, cell proliferation and migration were analyzed in MDA-MB-231, MCF-7, SKBR3 and BT474 cells. We tested the protein level in breast cancer cell lines and clinical samples (Supplementary Figure 2). Transient over expression of HOXA11, validated by qPCR (data not shown), suppressed cell proliferation in all four cell lines (Figure 3A) and slightly delayed wound closure in MDA-MB-231 cells (Figure 3B, *P*=0.1341). Unfortunately, no statistically significance was found in cell migration analyses in all four cell lines. Thus, HOXA11 suppressed cell proliferation but not cell migration in breast cancer cells.

### Low expression of HOXA11 is associated with poor prognosis

**Figure 4:**
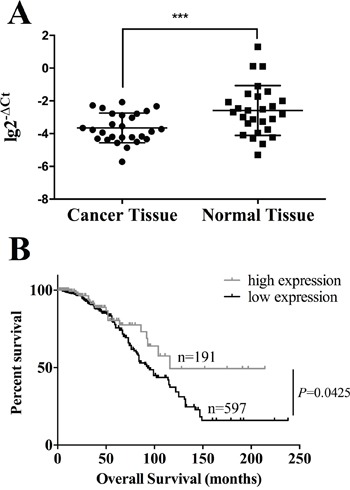
The high expression of HOXA11 prolongs overall survival in breast cancer patients **A**. The expression level of HOXA11 in 30 pairs of cancer and normal tissue. The average expression of HOXA11 is lower in cancer tissue than in normal tissue (P=0.0007). **B**. Kaplan-Meier curves show that patients with high expression of HOXA11 had a significantly worse prognosis than those with low expression of HOXA11 (P=0.0425, log-rank test).

DNA methylation is a key mechanism in gene silencing. We evaluated the mRNA expression level of HOXA11 in another 30 pairs of breast cancer and normal tissues which were randomly chosen from the hospital's biobank. The average expression level of HOXA11 in cancer tissues was significantly lower than in normal tissues (Figure 4A). Since we did not have HOXA11 expression data of the cohort employed in methylation analyses, we accessed TCGA database to investigate the correlation between HOXA11 mRNA expression and overall survival. As we expected, the survival probability of HOXA11 high expression group was markedly higher than of HOXA11 low expression group (Figure 4B, *P*=0.0425), indicating that low expression of HOXA11 is associated with poor prognosis.

## DISCUSSION

HOXA11, as a member of HOX family, is essential in development [13] and is required in endometrial receptivity and implantation [14, 15]. Abnormal HOXA11 methylation has been implicated in breast cancer [16–18]. However, the influence of HOXA11 hypermethylation on tumorigenesis is not clear.

In this study, we evaluated HOXA11 methylation level in paired breast cancer and normal tissues as well as in matched serum samples collected from 264 Chinese breast cancer patients. The methylation rate of HOXA11 is significant higher in cancer tissue (45.08%) than in paired normal tissue (6.82%) and matched serum specimens (16.36%). Circulating cfDNA released from cancerous tissues into blood reflects the methylation profiles in tumor cells and can be employed as a diagnostic biomarker in breast cancer [19]. The methylation rate of HOXA11 in serum is lower than in matched cancer tissues. According to this result, the alternative causes include cfDNA fragmentation and methods with impaired sensitivity. Although HOXA11 methylation solely is under quantified as a biomarker in serum, it can be combined with other hypermethylated genes to sever as biomarkers to distinguish cancer and normal tissues in non-invasive examination.

We noticed statistically significant correlations between HOXA11 hypermethylation and positive family history, IDC, positive lymph node metastasis and positive P53 staining (Table 1). First, 30 out of 264 cases have a documented family cancer history and 19 out of these 30 cases were defined as HOXA11 hypermethylated. Additionally, among patients with family cancer histories, the cases with hypomethylation of HOXA11 survived longer than those with hypermethylation (Supplementary Figure 3). The heritability of DNA methylation [20] might be the underlying link between HOXA11 hypermethylation and positive family history. Second, 1 out of 26 ductal carcinoma *in situ* (DCIS) cases has hypermethylatoin of HOXA11 meanwhile 118 out of 238 IDC cases show a high level of methylation. Considering the dynamics of HOXA11 methylation in development, this result infers the potential role of HOXA11 in breast cancer initiation and promotion. Third, HOXA11 prefers to be hypermethylated in cases with lymph node metastasis. This is consistent with the reports of other cancers [21, 22] and indicates a potential role of HOXA11 in metastasis. Last, the hypermethylation of HOXA11 is more frequently detected in P53 positive group. Another study of methylation patterns in breast tumors finds out that HOXA11 is highly methylated in P53 wild type groups [18]. The relationship between IHC expression of P53 and the mutational status of the TP53 gene is equivocal [23]. Different cohorts and methods make these two results incomparable. Further studies might be required in understanding the connection between P53 and HOXA11.

The hypermethylation of HOXA11 is an unfavorable prognostic biomarker in several cancers, especially in the female hormone dependent cancers such as ovarian cancer [7] and endometrial adenocarcinoma [21]. Besides, HOXA11 hypermethylation is also an independent predictor in breast cancer (Figure 1B), Table 2. By accessing the TCGA breast cancer database, we discovered that patients with HOXA11 low expression have short overall survival time (Figure 4B). Although it would be more convinced if we had the data of methylation status and expression level of HOXA11 from the same cohort, our results validate the clinical utility of HOXA11 as a biomarker in breast cancer.

HOXA11 expression was restored by 5-azadC, a demethylation reagent, in breast cancer cell lines in our experiment as well as in previous study [10]. In addition, this phenomenon was also described in other malignancies [8, 22]. Hence, DNA methylation alone is sufficient to silence HOXA11 in tumors. This universal demethylation reagent re-expresses numbers of genes including HOXA11 and enhances chemosensitivity in breast cancer [24]. We propose that HOXA11 might be a potential therapeutic target. In order to investigate the function of HOXA11 in breast cancer, we employed cell proliferation assay and observed an inhibition effect in all analyzed cell lines (Figure 3A). The similar response has been discovered in non-small cell lung cancer [8] and gastric cancer [22]. Based on previous studies and our results, HOXA11 is a potential tumor suppressor in breast cancer.

However, the underlying mechanism of HOXA11's anti-tumor activity is unclear. Previous study shows that HOXA11 might inhibit gastric cancer through Wnt signaling pathway [22]. Moreover, non-coding RNAs were deemed to exert negatively regulation in HOXA11 expression [15, 25]. To find out the genes related to HOXA11, we performed PCR microarray assay. The personalized PCR microarray contains a panel of genes related to breast cancer tumorigenesis and development. MDA-MB-231 cells were employed in this assay due to the inhibition of proliferation and migration by HOXA11 overexpression. All genes except ZNF703 and SNAI1 in the microarray panel were upregulated after HOXA11 plasmid transfection (Supplementary Figure 4). ZNF703 was identified as the driver of 8p12 locus amplication in breast cancer development [26]. Snail encoded by SNAI1 plays a role in recurrence of breast cancer by downregulating E-cadherin and inducing an epithelial to mesenchymal transition [27]. Thus, the down regulation of ZNF703 and SNAI1 by HOXA11 overexpression may contribute to breast cancer suppression. Further studies are warranted to discern the role of HOXA11 in breast cancer.

In conclusion, the highly methylation of HOXA11 is associated with invasive ductal carcinomas, cases with positive family cancer history, patients with lymph nodes metastasis and P53 positive cases. Hypermethylation of HOXA11 and low expression of HOXA11 also indicate poor prognosis. HOXA11 methylation correlates inversely with its expression. Over expression of HOXA11 inhibits cell proliferation in four breast cancer cell lines. Our finding suggests that HOXA11 can be applied as a clinical biomarker of prognosis and is a potential tumor suppressor in breast cancer.

## MATERIALS AND METHODS

### Subjects and sample collection

Cases for this study came from histologically confirmed breast cancers initially diagnosed between 2009 and 2011 at the Harbin Medical University Cancer Hospital. A total of 264 female breast cancer patients with ages ranging from 21 to 77 years (47.3 years on average) were enrolled in this research and none of them received anti-tumor therapies prior to surgery. A median 60-month follow-up was conducted to all patients, from whom informed consent was obtained. The study complied with the approval institutional guidelines (the Ethical Committee of the Harbin Medical University). The pathological tumor staging was assigned according to the American Joint Committee on Cancer Tumor-Node-Metastasis Classification. Complete clinical and follow-up information was available for all patients.

All specimens (∼0.125 mm^3^) including breast cancer tissues and paired normal tissues (at least 30 mm from malignant lesions) were snap-frozen in -80°C freezer within one hour after mastectomy. Matched blood samples were obtained from 264 patients before surgery. Serum was separated by centrifugation within 2 hours after collection and stored at -80°C until use. DNA was extracted from all samples including serum, and was used in a MethyLight assay.

### Molecular subtype classification of breast cancer

All tissue sections of breast cancer specimens were routinely processed in immunohistochemistry (IHC) assays for detection of estrogen receptor (ER), progesterone receptor (PR), human epidermal growth factor receptors 2 (HER2), Ki-67 and P53. Fluorescent *in situ* hybridization (FISH) was employed only if HER2 amplification was inconclusive in IHC staining. ER and PR staining scores were considered positive if there are at least 1% positive tumor nuclei in the sample [28]. The standard of HER2 positive was based on the percentage of the membrane staining of tumor cells and the value of a FISH ratio. A positive HER2 result is IHC staining of 3+ or a FISH ratio of more than 2.2 [29]. Cells stained for Ki-67 and P53 were counted and represented as a percentage. Low expression was considered as a Ki-67 index of lower than 14% [30] and P53 of not higher than 25% [31].

Molecular subtypes of breast cancer were classified according to the criteria approved by The 12th St Gallen International Breast Cancer Conference (2011) Expert Panel [32]. The detailed criteria were as follows: Luminal A type, ER- and/or PR-positive and HER2-negative and low Ki-67 index (< 14%); Luminal B type, (HER2-negative) ER- and/or PR-positive and HER2-negative and high Ki-67 index (≥ 14%); (HER2-positive) ER- and/or PR-positive and HER2 over-expressed or amplified and any Ki67 index; HER2-positive type, ER- and PR-negative and HER2 over-expressed or amplified; Triple-negative breast cancer (TNBC) type, ER-, PR- and HER2-negative.

### Cell culture

MDA-MB-231 and MCF-7 cells were cultured in 37°C incubator with 5% CO_2_ and maintained in DMEM (Lonza, Walkersville, MD, USA) containing 5% fetal bovine serum (ATCC, Rockville, MD, USA), 100 IU/ml of penicillin and 100 μg/ml streptomycin (ATCC, Rockville, MD, USA). SKBR3 and BT474 cells were cultured in 37°C incubator with 5% CO2 and maintained in RPMI (Lonza, Walkersville, MD, USA) containing 5% fetal bovine serum (ATCC, Rockville, MD, USA), 100 IU/ml of penicillin and 100 μg/ml streptomycin (ATCC, Rockville, MD, USA).

### DNA extraction and bisulfite modification

Genomic DNA (gDNA) was isolated from 264 pairs of primary breast tumors and corresponding normal tissues using an AxyPrepTM Multisource Genomic DNA Miniprep Kit (Axygen Scientic, San Francisco, CA, USA) following the manufacturer's instructions. Serum cell-free DNA (cfDNA) was obtained from 1 ml of serum by QIAamp Circulating Nucleic Acid Kit (Qiagen, Hilden, Germany) following the manufacturer's protocol. DNA quantification was determined by a spectrophotometer (Gene Quant Pro, Amersham Biosciences, England). The gDNA of cell lines was extracted by phenol-chloroform extraction followed by ethanol precipitation and the concentration was determined by NanoDrop 2000 (Thermo Scientific, Hudson, NH, USA).

For clinical samples, 500 ng of gDNA and 100 ng of cfDNA were applied for bisulfite conversion using the EZ DNA Methylation-Gold kit (Zymo Research, Orange, CA, USA) following the manufacturer's protocol. For cell lines, 1 μg of gDNA was diluted in water to a final volume of 50 μl. To create single-strand DNA, the samples were incubated with 5.5 μl of 2M NaOH at 37°C for 10 minutes, and then at room temperature for another 10 minutes. Freshly prepared 30 μl of 10mM hydroquinone (Sigma-Aldrich, St Louis, MO, USA) and 520 μl of 3M sodium bisulfite (Sigma-Aldrich, St Louis, MO, USA) at pH 5.0 were added to each reaction tubes. After 16 hours of incubation in dark at 50°C, DNA was desalted by Wizard DNA clean-up system (Promega, Madison, WI, USA) and reconstituted in 50 μl of distilled water. Then the samples were incubated with 5.5 μl of 3M NaOH for 5 minutes at room temperature to complete the conversion of unmethylated cytosine to uracil. Bisulfite treated DNA was purified and concentrated by ethanol precipitation and stored at -80°C until use.

### MethyLight assay

Clinical gDNA and cfDNA treated with sodium bisulfite were analysed by MethyLight, a fluorescence-based, real-time quantitative PCR (qPCR) assay, as described previously [33]. TaqMan Minor Groove Binder (Applied Biosystems, Foster City, CA, USA) PCR was performed with primers specific for the bisulfite-converted sequence. Globin was used as an internal reference gene. The primers and probe for globin were as follows: forward primer, 5′-AGGTAGAAAAGGAGAATGAAGATAAA-3′; reverse primer, 5′-CTTTCCACTCTTTTCTCATTCTCTC-3′; product size, 143 bp; probe sequence, 5′-AGGAGGATAAGGAAGAGGGGAAATAGG-3′. The set of primers and probe for HOXA11 was as follows: forward primer: 5′-GTTGTTGGCGGTTTAGGGAC-3′; reverse primer: 5′-GCCTCTACCTCCGACCCTAA-3′; product size is 167 bp; probe sequence: 5′-AGAGTGTAATTAAGTTATCGTGTA-3′. For each PCR reaction, 2.5 mM MgCl_2_, 10 μM dNTP, 0.25 μM forward and reverse primers, 0.1 μM probe, 1× Platinum Taq buffer and 0.5 unit of Platinum Taq polymerase (Invitrogen, Carlsbad, CA, USA) were used in a total volume of 10 μl. PCR was performed under the following conditions: 95°C, 3 min; followed by 45 cycles of 95°C for 10 s and 60°C for 30 s. Cycle threshold (Ct) values obtained in PCR analyses were used as a measure of the degree of methylation at the analyzed locus. Relative quantification was performed based on the threshold cycles of HOXA11 and internal reference gene (globin). The value of methylation at a specific locus was calculated by the 2^-ΔΔCt^ method [34], where ΔΔCt = (Ct _(HOXA11)_ - Ct_(globin)_) _cancer_ - (Ct _(HOXA11)_ - Ct_(globin)_) _normal_. The cut-off value of ≥ 1.5 [35] was delineated as hypermethylation in cancer tissue. In normal tissue, the gene of interest was considered unmethylated if its Ct value was ≥ 40. The relative methylation level in serum was calculated by the 2^-ΔCt^ method, where ΔCt = Ct _(HOXA11)_ - Ct_(globin)_ [36]. All amplification efficiencies were similar. We tested each sample in triplicate.

### Methylation specific PCR (MSP)

MSP used to determine the methylation status of HOXA11 promoter region was performed as previously described [37]. MSP amplification was carried out with the following reaction mixture: 10 μl of PCR master buffer (GeneDEPOT, Barker, TX, USA), 1 μl of Taq DNA polymerase (GeneDEPOT, Barker, TX, USA), 1 μl of 10 μM methylated or unmethylated primers, 50 ng of template DNA and distilled water brought the final volume to 20 μl. Genomic DNA methylated by M.SssI (New England Biolabs, Beverly, MA, USA) was used as a methylated control and peripheral lymphocyte DNA modified by sodium bisulfite was used as a unmethylated control. The methylated primers were as follows: forward primer, 5′-GTTTACGGTGTTATAGAAATTGGAC-3′; reverse primer, 5′-GTACACAAAAACTACCTACAAACGC-3′; product length, 129bp. The unmethylated primers were as follows: forward primer, 5′-TTTATGGTGTTATAGAAATTGGATGA-3′; reverse primer, 5′-TCATACACAAAAACTACCTACAAACAC-3′; product length, 130bp [12]. A total of 35 cycles were run with an annealing temperature of 55°C. PCR products were visualized after electrophoresis on 2% agarose (Sigma-Aldrich, St Louis, MO, USA) gels staining with ethidium bromide (Thermo Scientific, Hudson, NH, USA).

### 5-aza-2’-deoxycytidine (5-azadC) treatment

For 5-azadC (TCI America, Portland, OR, USA) treatment, MDA-MB-231 and MCF-7 cells were grown in a 6-well plate at low density and treated with 20μM of 5-azadC dissolved in distilled water for 72 hours [38]. The culture medium with 5-azadC was refreshed every 12 hours. Cells without treatment were used as a control.

### HOXA11 mRNA expression

The total RNA of breast cancer cell lines was extracted with E.Z.N.A. total RNA kit (Omega Bio-tek Inc., Norcross, GA, USA). Reverse transcription (Quanta Biosciences, Gaithersburg, MD, USA) followed by qPCR (BIOLINE, Taunton, MA, USA) was employed to determine the mRNA expression of HOXA11. QPCR was performed under the following conditions: 95°C, 3 min; followed by 40 cycles of 95°C for 30 s, 58°C for 30 s and 72°C for 30s; 72°C for 7 minutes and 4°C hold. The set of primers was as follows: forward primer, 5′-CGGCAGCAGAGGAGAAAG-3′; reverse primer, 5′-TATAGGGGCAGCGCTTTT-3′; product length, 132 bp.

### Transient transfection

HOXA11 over expression plasmid shared by Dr. Wagner at Yale [39] was used in transient transfection with FuGENE 6 (Promega, Madison, WI, USA). The expression level of HOXA11 after transfection was determined with qPCR. MTT assay and wound healing assay were performed 24 hours post-transfection.

### MTT assay

MDA-MB-231 cells, MCF-7, SKBR3 and BT474 cells were seeded into 96-well plate at 2,500 cells and 5,000 cells per well, respectively. Transient transfection was performed on day-1 (24 hours prior to MTT assay). To measure cell proliferation, the cells were incubated with MTT reagent (AMRESCO, Solon, OH, USA) for 2 to 4 hours on day 0, day 1, day 2, day 4 and day 6. The absorbance at 570nm was recorded on the indicated days. Each assay was performed in triplicate.

### Wound healing assay

HOXA11 over expressed group and control group were seeded into 24-well plate and cultured overnight. A scratch wound was created with a sterile 200 μl tip in each well when cell confluence reached 100%. Microscopic photography was taken right after scratching and 48 hours later. The cell migration area was measured with ImageJ (NIH, Bethesda, MD, USA). Each assay was performed in triplicate.

### Western blot

The total protein from frozen tissues and breast cancer cell lines was purified using RIPA Buffer. Per sample, 30mg of protein was loaded into a denaturing polyacrylamide gel containing SDS and transferred to a metha- nol-activated PVDF filter membrane (Bio-Rad, Hercules, CA, USA). Before immunodetection, membranes were blocked with 5% non-fat dry milk. Primary antibodies, anti-HOXA11 (1:300; Cat#ab72591, Abcam, Cambridge, MA, USA) were diluted in the blocking buffer and incubated at 4 overnight. After subsequently washing with TBST, the membranes were incubated with secondary antibody for 2 hours at room temperature. Beta-actin (Cat#TA-09, ZSGB-Bio) was used as internal reference protein. The experiment was repeated in triplicate. The bands were visulized by enhanced chemiluminescence detection reagents (Applygen Technologies Inc., Beijing, China).

### PCR microarray

Total cellular RNA was purified from MDA-MB-231 transfected with HOXA11 overexpression plasmid, harvested at 24h and 48h, and compared on microarray against mock transfected control. cDNA was generated by ReverTra Ace qPCR RT Kit (TOYOBO, Shanghai, China). The customized arrays used in the experiments were manufactured by QIAGEN (CAPH12267, QIAGEN, Valencia, CA, USA). Each sample was performed in triplicate. The data was analyzed by R.

### Analyse data in TCGA (The cancer genome atlas) database

In order to review the association between HOXA11 expression and overall survival, we accessed TCGA breast invasive carcinoma gene expression database (RNAseq, IlluminaHiSeq, V2, Feb 24, 2015) by cancer browser at https://genome-cancer.ucsc.edu/ In model of a binary variables, we interpreted mRNA expression of HOXA11 as high expression if the value is above 4.5, and as low expression if the value is under 4.5. By these criteria, 191 cases were classified as HOXA11 high expression and 597 cases were defined as HOXA11 low expression.

### Statistical analysis

Statistical analyses were performed by IBM SPSS software 20.0 (IBM Corp., Armonk, NY, USA) and GraphPad Prism 6.0 (GraphPad Software Inc., La Jolla, CA, USA). Chi-square test was used to detect differences in clinical data and Student's t-test was employed for experimental data. Kaplan-Meier survival curves using log-rank statistics was used to evaluate the association of overall survival and the expression or methylation of HOXA11. Cox multivariate regression analyses were performed to evaluate the influence of different variables on survival. Only variables with P<0.1 in the univariate analysis were included in the multivariate model. Risk ratios and their 95% confidence intervals were recorded for each marker. A P-value less than 0.05 was considered statistically significant for all analyses.

## SUPPLEMENTARY MATERIALS FIGURES AND TABLES


