# Supplementary material for: Homeobox A11 hypermethylation indicates unfavorable prognosis in breast cancer
Source: Oncotarget. 2016 Dec 25;8(6):9794–805. doi: 10.18632/oncotarget.14216 (PMC5354771; doi:10.18632/oncotarget.14216)
Supplement: Supplementary file 1 [file oncotarget-08-9794-s001.pdf]

# Homeobox A11 hypermethylation indicates unfavorable prognosis in breast cancer

## SUPPLEMENTARY FIGURES AND TABLE

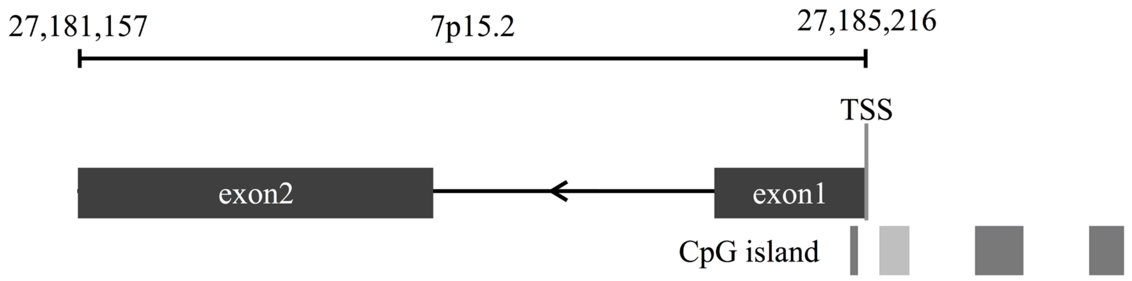


**Supplementary Figure 1: Schematic diagram of HOXA11 gene.** Grey blocks indicate CpG islands in the promoter region of HOXA11 gene and light grey block represents the CpG island targeted by MethyLight and MSP. TSS, transcription starting site.

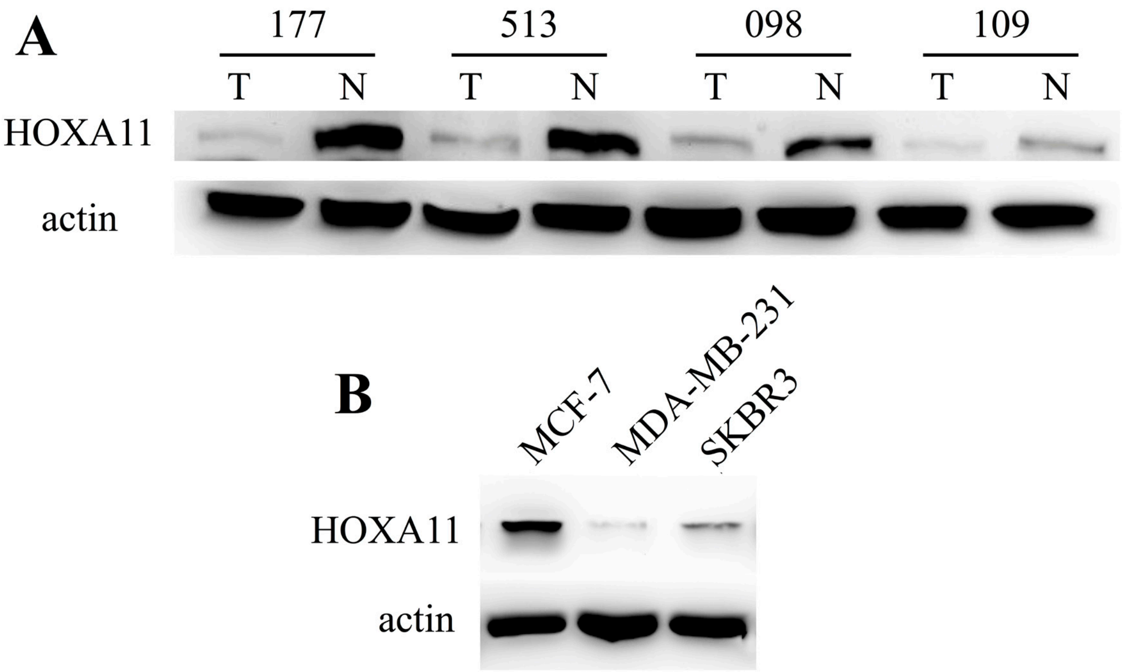


**Supplementary Figure 2: The protein expression level of HOXA11 in breast cancer tissues and cell lines. A.** HOXA11 protein expression in clinical specimens. T indicates tumor tissue and N indicates matched normal tissue. The numbers indicate sample No. in our hospital's biobank. **B.** HOXA11 protein expression in breast cancer cell lines.

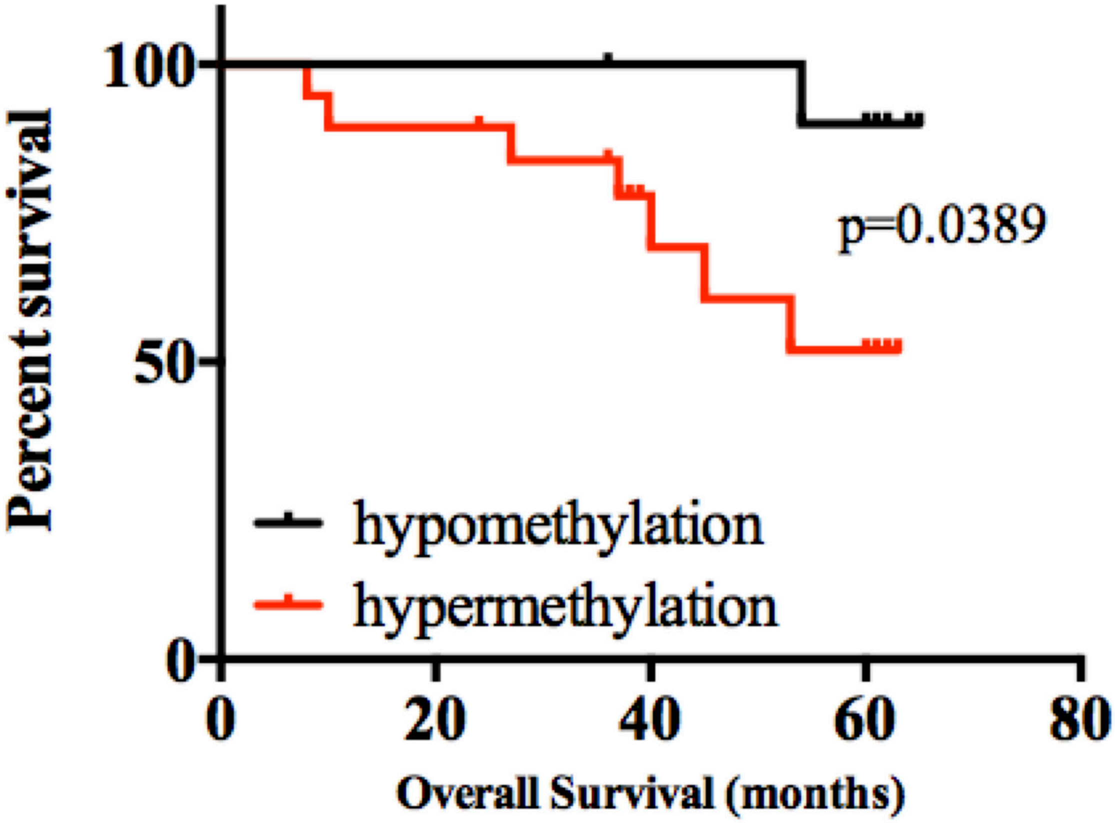


**Supplementary Figure 3: The survival curve of patients with a family cancer history.**

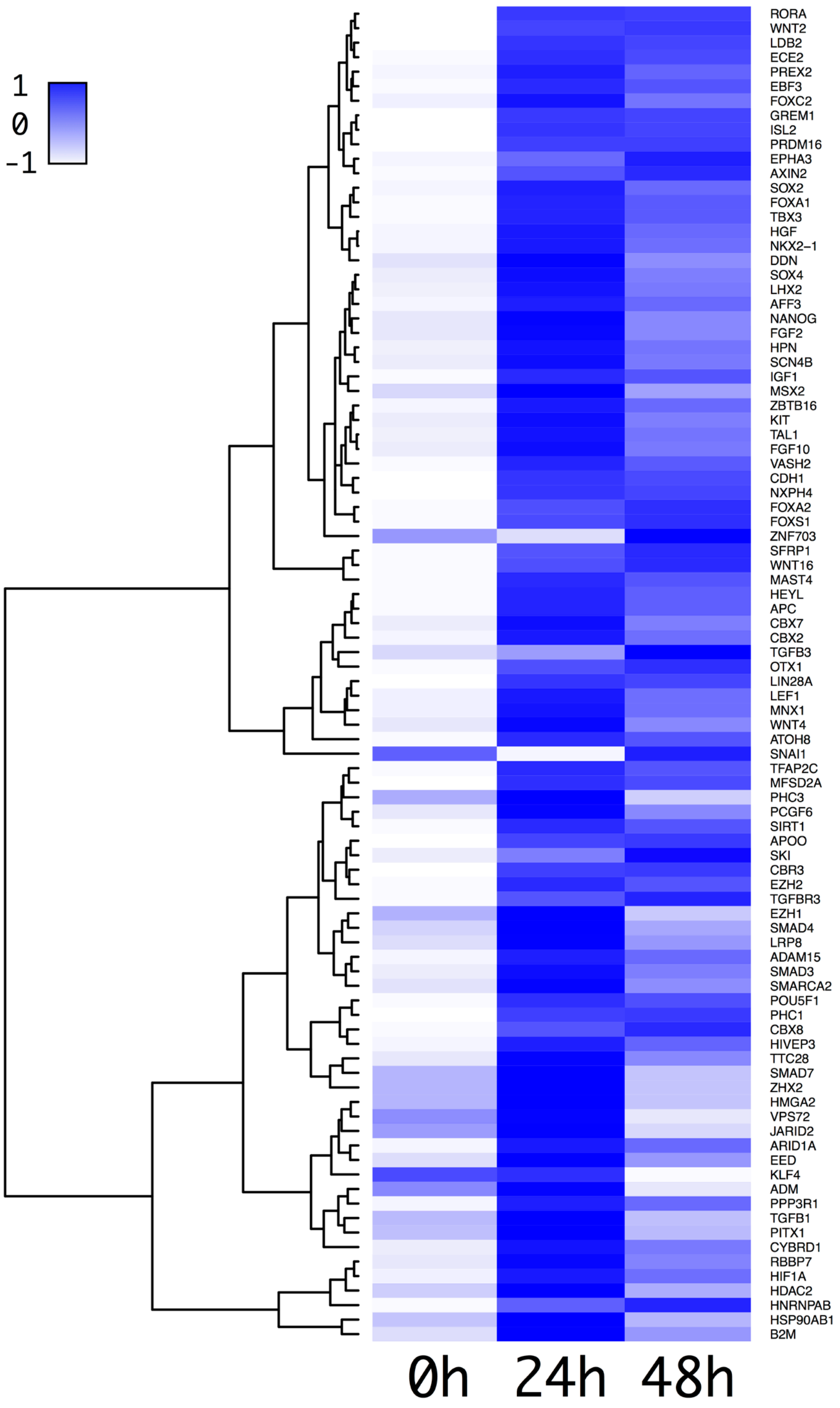


**Supplementary Figure 4: PCR microarray.** The personalized PCR microarray contains a panel of genes related to breast cancer tumorigenesis and development. MDA-MB-231 cells transfected with HOXA11 over expression plasmid, harvested at 24h and 48h, and mock transfected control were analyzed in this assay. Each sample was performed in triplicate.

**Supplementary Table 1: Prognostic factors in univariate analysis**

| | Univariate |
|---|---|
| Variables | P value |
| Age (years) | 0.613 |
| Tumor size (cm) | <0.001 |
| LNM | 0.002 |
| TNM Stage | 0.003 |
| Histological grade | <0.001 |
| ER status | 0.438 |
| PR status | 0.518 |
| HER2 status | 0.087 |
| Ki67 status | 0.565 |
| P53 status | 0.872 |
| HOXA11 methylation | <0.001 |

Abbreviations: CI = confidence interval; ER = oestrogen receptor; LNM = lymph node metastasis; PR = progesterone receptor; TNM = tumour node metastasis.
